# Stigmasterol alleviates allergic airway inflammation and airway hyperresponsiveness in asthma mice through inhibiting substance-P receptor

**DOI:** 10.1080/13880209.2023.2173252

**Published:** 2023-02-14

**Authors:** Jimei Zhang, Chonghong Zhang, Li Miao, Zimin Meng, Ning Gu, Guifang Song

**Affiliations:** a Department of Material supply, the Affiliated Yantai Yuhuangding Hospital of Qingdao University, Yantai, Shandong, China; b Department of Cardiology, First Ward, Yantai Yeda Hospital, Yantai, Shandong, China; c Department of Cardiovascular medicine, Weihai Municipal Hospital, Weihai, Shandong, China; d Department of Cardiology, the Affiliated Yantai Yuhuangding Hospital of Qingdao University, Yantai, Shandong, China

**Keywords:** Inflammation response, oxidative stress, mucus hypersecretion

## Abstract

**Context:**

Stigmasterol has significant anti-arthritis and anti-inflammatory effects, but its role in immune and inflammatory diseases is still unclear.

**Objective:**

The potential advantages of stigmasterol in asthma were explored in IL-13-induced BEAS-2B cells and asthmatic mice.

**Materials and methods:**

The optimal target of stigmasterol was confirmed in asthma. After detecting the cytotoxicity of stigmasterol in BEAS-2B cells, 10 μg/mL and 20 μg/mL stigmasterol were incubated with the BEAS-2B cell model for 48 h, and anti-inflammation and antioxidative stress were verified. Asthmatic mice were induced by OVA and received 100 mg/kg stigmasterol for 7 consecutive days. After 28 days, lung tissues and BAL fluid were collected for the following study. To further verify the role of NK1-R, 0.1 μM WIN62577 (NK1-R specific antagonist), and 1 μM recombinant human NK1-R protein were applied.

**Results:**

NK1-R was the potential target of stigmasterol. When the concentration of stigmasterol is 20 μg/mL, the survival rate of BEAS-2B cells is about 98.4%, which is non-toxic. Stigmasterol exerted anti-inflammation and antioxidant stress in a dose-dependent manner and decreased NK1-R expression in IL-13-induced BEAS-2B. Meanwhile, *in vivo* assay also indicated the anti-inflammation and antioxidant stress of stigmasterol after OVA challenge. Stigmasterol inhibited inflammation infiltration and mucus hypersecretion, and NK1-R expression.

**Discussion and Conclusions:**

The protective effect of stigmaterol on asthma and its underlying mechanism have been discussed in depth, providing a theoretical basis and more possibilities for its treatment of asthma.

## Introduction

Asthma is a chronic inflammatory disease, which is characterized by airway hyperresponsiveness and involves many inflammatory cells and mediators (Ward et al. 2005). Substance P is present in sensory afferent nerves and inflammatory cells of the airway. A large amount of stimuli (e.g., allergen, ozone) could release it (Joos et al. 2001). An increased level of substance P was found in the sputum and bronchoalveolar lavage fluid of asthmatic patients (Nieber et al. 1992; Tomaki et al. 1995). Abnormal levels of substance P resulted in smooth muscle contraction, vasodilation, increased vascular permeability, activation of inflammation cells (mast cells, B and T lymphocytes, and macrophages), submucosal gland secretion, the chemical attraction of eosinophils and neutrophils, vascular adhesion of neutrophils (Joos et al. 2001). Substance P (SP), also known as neurokinin-1 (NK-1), is an 11-amino acid neuropeptide of the tachykinin family, which is synthesized by neutral tissues and preferentially activates substance P receptor (Neurokinin-1 Receptor, NK1R). NK1R is primarily involved in neurogenic inflammation: mucus secretion and microvascular leakage (Maggi 2000). Substance P and its receptor NK1R have been considered to be important players in human respiratory diseases (asthma and chronic obstructive diseases etc.).

Stigmasterol, a naturally available sterol, is one of a larger class of plant compounds, phytosterols (Moreau et al. 2002), that is widely present in plant-derived food (Ryan et al. 2007) and common medicinal plants all over the world (Zheng 1994). Phytosterols have more biological benefits including anticancer (Bradford and Awad 2007; Shahzad et al. 2017), anti-allergic effect (Antwi et al. 2017), anti-inflammation (Rocha et al. 2016; Vilahur et al. 2019), and lipid-lowering (Dumolt and Rideout 2017; Makhmudova et al. 2021). Although stigmasterol has significant anti-arthritis and anti-inflammatory effects (Gabay et al. 2010; Chen et al. 2012), its role in immune and inflammatory diseases (such as asthma) is still unclear. A study only revealed the anti-airway inflammation of stigmasterol in a guinea pig model of ovalbumin (OVA)-induced asthma (Antwi et al. 2017), but did not reveal its related target and mechanism.

This study explored the potential advantages of stigmasterol in asthma and its underlying molecule target. We assessed the anti-inflammation and anti-allergic effect of stigmasterol in OVA-induced asthma mice and IL-13-induced BEAS-2B cells, and investigated if NK1R is the underlying target of stigmasterol in the treatment of asthma.

## Materials and methods

### Molecular docking method

The 3D molecular structure of stigmasterol is downloaded from PubChem database (https://pubchem.ncbi.nlm.nih.gov/) and imported into UCSF Chimera, and then finally converted into PDB format. PDB format of NK1R was downloaded from PDB (https://www.rcsb.org/) database, and imported into Chimera in order to delete non-standard receptors followed by protein optimization *via* AutoDock Vina, and the file was saved as pdb format. Finally, molecular docking between stigmasterol and NK1R was performed with AutoDock Vina software.

### Cell incubation

BEAS-2B cells were purchased from Pricella (CL-0496) and incubated in DMEM medium containing 10% FBS and 1% penicillin-streptomycin in an atmosphere of 5% CO_2_ at 37 °C. The complete medium was replaced every 2 days. When cell confluence reached 80% approximately, cells were digested with trypsin and subcultured.

### MTT assay

At the beginning of the experiment, we first verified the toxicity of the drug to BEAS-2B cells by MTT. Cells were seeded into 96-wells plate, and then different concentrations (0, 1, 5, 10, 20, 40, 60 μg/mL) of stigmasterol were added into the wells for 48 h. Then, 200 μL MTT solution (180 μL DMEM medium without FBS + 20 μL 5 mg/mL MTT) was added to the plates for 4 h. Then, MTT solution was removed and 150 μL DMSO was added, and plates were shaken at low temperature for 10 min in order to fully dissolve the crystallization. Finally, a microplate reader was used to detect absorbance values at 490 nm. Cell viability was calculated according to the following formula.

$$\mathrm{Cell}\;\mathrm{viability}\;(\%)=\frac{{\mathrm{OD}}_{\mathrm{treated}}-{\mathrm{OD}}_{\mathrm{zero}}}{{\mathrm{OD}}_{\mathrm{control}}-{\mathrm{OD}}_{\mathrm{zero}}}\times 100$$

OD_treated_: cells were incubated with stigmasterol; OD_control_: cells were incubated without stigmasterol; OD_zero_: cells were only treated with culture medium.

### Cell administration

***Part 1***: To verify the effect of stigmasterol on IL-13-induced BEAS-2B cells, and the regulation of stigmasterol in NK1R. BEAS-2B cells were stimulated by 20 ng/mL recombinant human IL-13 (ab270079, Abcam) for 72 h. After induction, cells were divided into 3 groups: IL-13 group, two concentrations of stigmasterol groups (Stig-L and Stig-H). We selected 10 μg/mL and 20 μg/mL stigmasterol to treat cells for 48 h. Cells were not treated with IL-13 in the control group.

***Part 2:*** To further confirm the underlying mechanism of NK1R as a potential target. We applied NK1R-specific antagonist WIN62577 (Sigma, CAS#: 138091-43-7) and agonist (recombinant human NK1R protein, ab152721, Abcam). After IL-13 stimulation, cells were grouped into 4 groups: IL-13 group, Stigmasterol group, WIN62577 group, and Stigmasterol + rh-NK1R protein (Stig-NK1R) group. In the Stigmasterol group and WIN62577 group, cells were treated with 20 μg/mL stigmasterol and 0.1 μM (Wei et al. 2018) WIN62577 for 24 h, respectively. In Stigmasterol + rh-NK1R group, cells were treated with 20 μg/mL stigmasterol and 1 μM (Zhang et al. 2021) recombinant human NK1R protein for 24 h.

### Flow cytometry

According to the experimental groups, we seeded cells with or without IL-13 into 6-well plates. After 24 h, cells were treated with different administrations for 24 h. Specific procedures of ROS were carried out according to the instructions of the kit (KGT010-1, Jiangsu KeyGEN BioTECH Corp., Ltd). DCFH-DA fluorescent probe was diluted with a serum-free medium at the ratio of 1:1000 and added into cells for 30 min. After washing 3 times with PBS, cells were resuspended in 0.5 mL PBS and detected by flow cytometry.

### Immunofluorescence staining

According to the design requirements of each group, the cells were fixed with 4% paraformaldehyde for 30 min after different treatments. 0.5% Triton X-100 was used to penetrate cells for 20 min and normal goat serum was used to block non-specific expression at room temperature for 30 min. Diluted primary antibody anti-neurokinin 1 receptor antibody (1:200, bs-0064R, Bioss) was added and incubated overnight at 4 °C. Next day, cells were incubated with secondary antibody Cy3-labelled goat anti-rabbit IgG (H + L) (A0516, 1:200, Beyotime) for 1 h. DAPI was diluted at a ratio of 1:200 and added into cells for 8 min. Finally, an anti-fluorescence quenching agent is used to seal the film to prevent fluorescence quenching. Fluorescence of the substance P antibody was observed under MF52-N fluorescent inverted microscope. Images were taken at the magnification of 200×.

### Western blot

Total proteins were extracted from BEAS-2B cells and mouse lung homogenates using TRIzol reagent. Bicinchoninic acid assay was performed to quantify protein concentration. Protein sample (30 μg) was separated by 10% sodium dodecyl sulphate-polyacrylamide gel electrophoresis and then shifted to PVDF membranes. After blocking by 5% non-fat milk for 1 h. The blots were incubated with primary antibody neurokinin-1 receptor polyclonal antibody (1:800, 17942-1-AP, Proteintech), GAPDH (1:5000, 10494-1-AP, Proteintech) at 4 °C overnight. Then, PVDF membranes were incubated with secondary antibodies (1:2000, ab6721, Abcam). After washing 3 times with TBST solution, a chemiluminescence (ECL) reagent was used to make proteins visible. The grey value of each band was recorded and analyzed by Image J 1.49p software.

### OVA-induced mice model of asthma and administration

Fifty male C57bl/6J mice (6 weeks old) were purchased from Jinan Pengyue Experimental Animal Breeding Co., Ltd. (Permission Number: SYXK(Lu)20190003). All animal procedures were performed according to the Declaration of Helsinki and the Guide for the Care and the Use of Laboratory Animals. All experiments in this study were supported by the Affiliated Yantai Yuhuangding Hospital of Qingdao University (Ethical number is 2022-195).

#### Model:

The specific methods of OVA-induced asthma mice were carried out as previously reported (Wang et al. 2016) with slight modification. Briefly, mice were randomly grouped into two groups: control and OVA groups. I prepared an immune injection (including 10 mg/mL OVA and Alum adjuvant). Mice in OVA group were sensitized by intraperitoneal injection of 100 μL immune injection (20 μg OVA) at days 0, 7 and 14. Three weeks after intraperitoneal injection of OVA, the mice were given a 20 μg/mL OVA by inhalation on days 21, 22, 23, 24, 25, 26, 27. In normal group, OVA was replaced by PBS. Asthma mice were divided into four groups: OVA group, Stigmasterol group, WIN62577 group, and Stigmasterol + rh-NK1R protein (Stig-NK1R) group.

#### Administration:

In corresponding groups, 100 mg/kg stigmasterol, 300 μg WIN62577, and 100 μg rh-NK1R were intraperitoneally injected into mice on days 21, 22, 23, 24, 25, 26, 27 before the challenge with OVA. Differential cell count (500 cells/slide) was performed in bronchoalveolar lavage fluid (BALF).

### Airway hyperreactivity detection

To evaluate airway hyperresponsiveness, invasive measures of dynamic lung resistance and compliance were measured by flexivent (SCIREQ, Emka Technologies) 2 days after the last drug injection. In short, the mice were anesthetized with 3% pentobarbital sodium (50 mg/kg), tracheotomized, and instantly intubated with a catheter (18-gauge) for mechanical ventilation. mechanical ventilation. The respiratory rate was set to 150 breaths/minute, the tidal volume was 0.2 mL, and 2 mL of H_2_O was applied as positive end-expiratory pressure. The pulmonary resistance (cm H_2_O mL/s) in each respiratory cycle was detected by the airflow signals after increased concentrations of methacholine (0 to 40 mg/mL).

### BALF collection and analysis

Mice were sacrificed after anesthesia with 3% pentobarbital sodium (50 mg/kg). BALF was collected. Briefly, trachea was inserted, lung tissues were then lavaged with PBS with 1% fetal calf serum and 0.5 mmol/L EDTA. After BALF centrifugation, cell pellets were resuspended in PBS containing 1% fetal calf serum. Total cell numbers in lung tissues were counted with a hemacytometer. Differential cell counts were implemented by cytospins stained with Diff-Quick. The levels of inflammatory cytokines IL-4, IL-5 and IFN-γ, and oxidative stress markers malondialdehyde (MDA), superoxide dismutase (SOD), catalase (CAT), glutathione (GSH) in the bronchoalveolar lavage fluid (BALF) and the supernatant of IL-13-induced BEAS-2B cells were measured by ELISA. Both BALF and cell medium were collected and centrifuged at 800 g for 10 min at 4 °C. Then, the supernatant was used for ELISA analysis. The experiment was carried out in strict accordance with the instructions. All kits were purchased from Nanjing Jiancheng Bioengineering Institute. IL-4 (H005), IL-5 (H006), IFN-γ (H025), MDA (A-003-1-2), SOD (A001-3-2), CAT (A007-1-1), GSH (A005-1-2).

### Pathological staining

After the mice were killed, part of the lung tissue was fixed in 4% paraformaldehyde, and the other part was stored at −80 °C. The fixed lung tissues were dehydrated and embedded in paraffin. Paraffin sections (3 μm) were dewaxed and rehydrated for hematoxylin and eosin (H&E) staining and periodic acid–Schiff reagent (PAS) staining. Procedures of PAS staining were carried out according to the kit instruction (G1281, Solarbio). We also analyzed the expression of NK1R in lung tissues by immunohistochemical staining. Primary antibody anti-neurokinin 1 receptor antibody was diluted at a ratio of 1:150. After staining, the infiltration of inflammatory cells and mucus secretion were observed under the microscope (100×). NK1R-positive expression was also observed and analyzed by Image J software.

### Statistical analysis

Statistical data were expressed as mean ± SD. Graphpad8.0.2 and SPSS19.0 are used for all data mapping and analysis. Statistical difference analysis in multiple groups was performed using ANOVA, followed by *Tukey*. P value less than 0.05 means significant difference between groups.

## Result

### Potential binding between stigmasterol and substance P receptor

**Figure 1.**
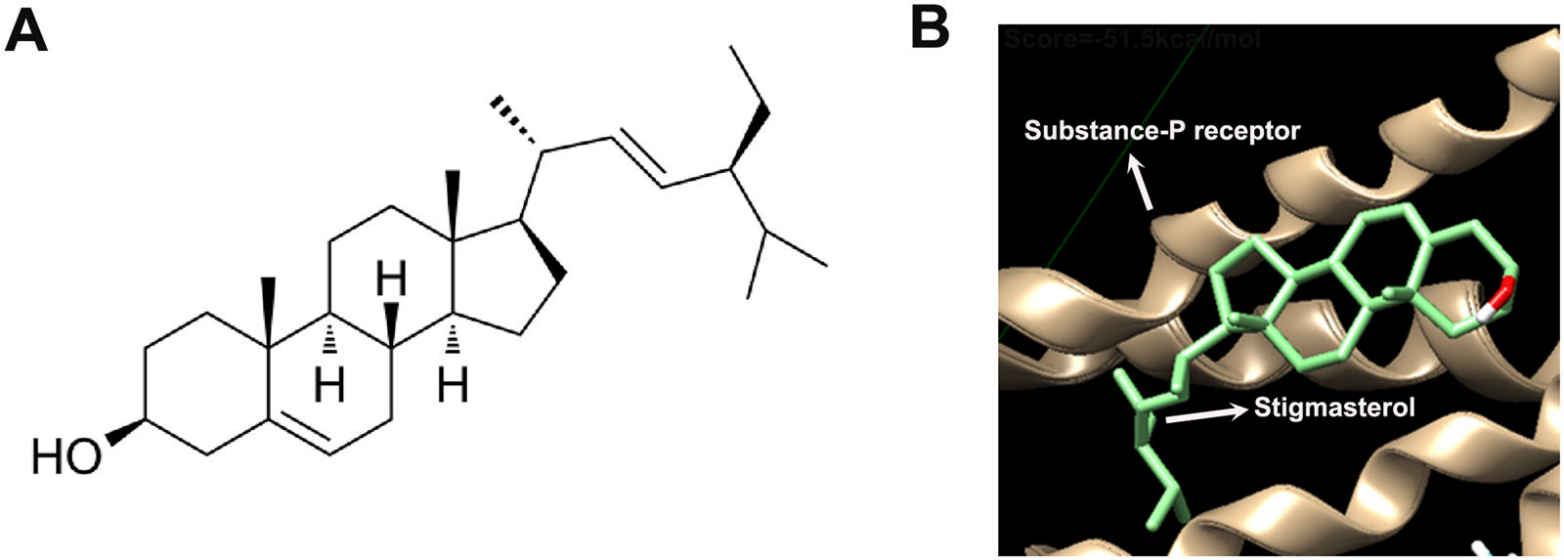
Potential combination between stigmasterol and Substance P receptor (NK1-R). (A) 2D chemical structure of stigmasterol; (B) AutoDock Vina docking technology was used to analyze the binding between stigmasterol and Substance P receptor.

The 2D chemical structure of stigmasterol was shown in Figure 1(A). We searched stigmasterol-related diseases, including asthma from TCMSP (https://old.tcmsp-e.com/molecule.php?qn=449). Meanwhile, we also found 34 targets related to asthma (https://old.tcmsp-e.com/disease.php?qd=77). In order to select a target molecule that binds more firmly to stigmasterol, we used AutoDock Vina docking technology to analyze the binding between stigmasterol and these 34 targets (Table 1). According to the scores, we selected the optimal target molecule, substance-P receptor. Molecular docking results showed that the score was −51.5 kcal/mol, and the absolute value was greater than 6, which confirmed that there was a stable binding between stigmasterol and substance-P receptor (Table 1 and Figure 1(B)). The above data suggest that substance P receptor may be a potential target of stigmasterol.

**Table 1.** Asthma-related targets and binding score given by AutoDock Vina.

Targets	Score
Adenosine A1 receptor	−7.4
Pyridoxal kinase	−8.8
Beta-1 adrenergic receptor	−8.2
Peroxisome proliferator-activated receptor gamma	−9.2
Arachidonate 5-lipoxygenase	−8.2
*Substance-P receptor*	*-51.2*
Leukocyte elastase	−8.5
CAMP-specific 3′,5′-cyclic phosphodiesterase 4B	−5.9
Integrin alpha-4	−7.9
Beta-2 adrenergic receptor	−10.1
Tumor necrosis factor	−7.1
Cysteinyl leukotriene receptor 1	−7.7
Rho-associated protein kinase 1	−6.1
Chymase	−7.0
E-selectin	−6.3
mRNA of Intercellular adhesion molecule-1	−5.4
Leukotriene C4 synthase	−10.4
Interleukin-5	−6.6
Tyrosine-protein kinase SYK	−7.6
Poly [ADP-ribose] polymerase-1	−7.4
Thromboxane-A synthase	−6.6
ADAM 33	−6.9
High affinity interleukin-8 receptor A	−7.3
Cytochrome P450 1A2	−9.6
Arginase-1	−7.4
Tumor necrosis factor receptor superfamily member 1 A	−7.2
Integrin beta-1	−7.8
1-Phosphatidylinositol phosphodiesterase	−7.6
Inhibitor of nuclear factor kappa B kinase beta subunit	−9.1
Tyrosine-protein kinase itk/tsk	−6.1
B2 bradykinin receptor	−5.5
Histamine H4 receptor	−8.9

### Stigmasterol inhibited inflammation response and oxidative stress, and reduced NK1-R expression in BEAS-2B cells induced by IL-13

**Figure 2.**
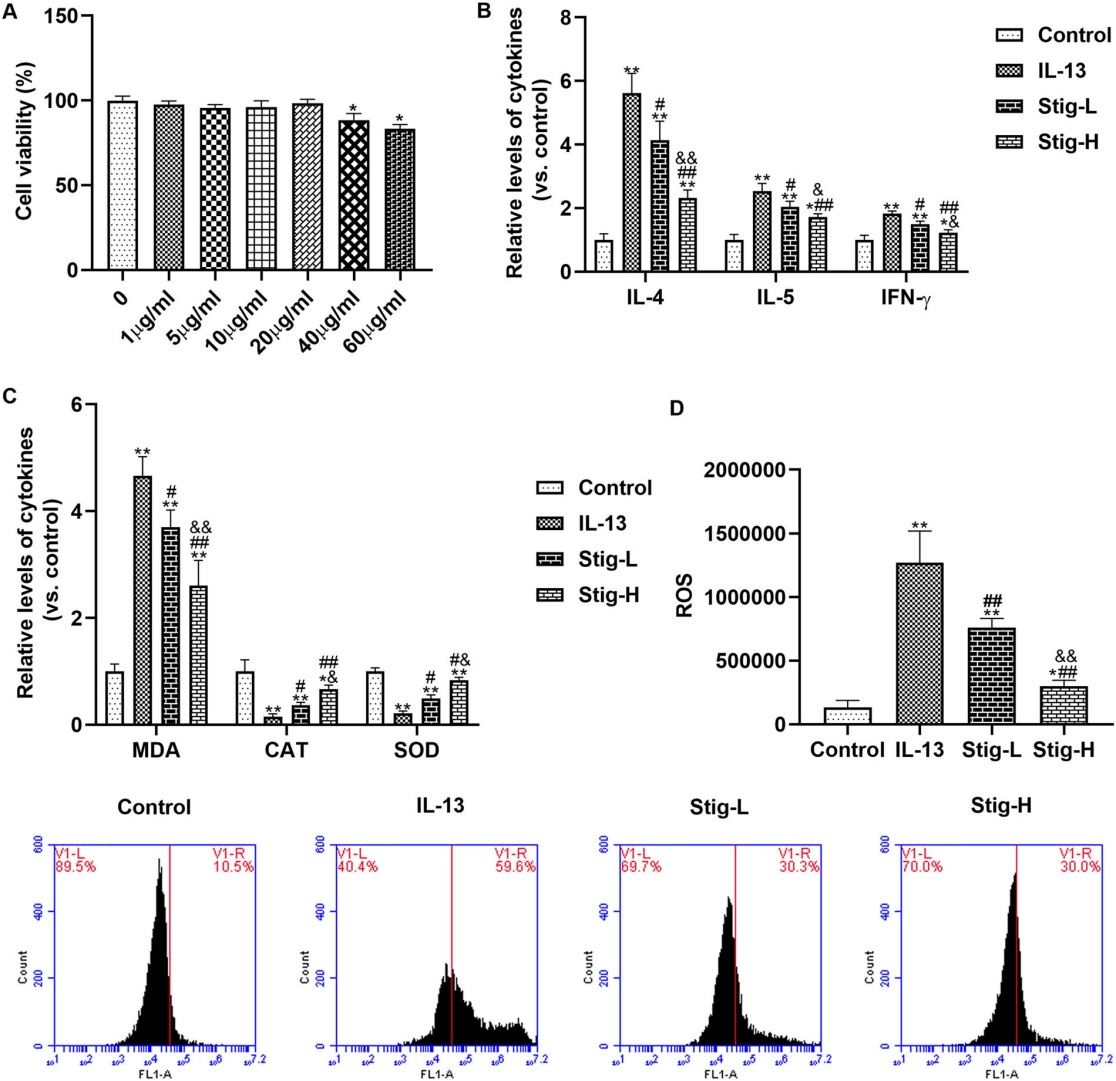
Stigmasterol inhibited inflammation response and oxidative stress by substance P receptor. (A) MTT was used to detect the effect of stigmasterol on the survival rate of BEAS-2B, so as to confirm whether stigmasterol has cytotoxicity to BEAS-2B cells. *p < 0.05. (B) After the induction of IL-13, inflammation factors IL-4, IL-5 and IFN-γ in cellular medium were measured by ELISA method. (C) Oxidative stress markers MDA, CAT and SOD in cell supernatant were measured by ELISA methods. (D) Level of ROS was detected by flow cytometry. We evaluated the effect of drugs on oxidative stress by oxidative stress markers and ROS levels. *p < 0.05, **p < 0.01, control; ^#^*p* < 0.05, ^##^*p* < 0.01, IL-13; ^&^*p* < 0.05, ^&&^*p* < 0.01, Stig-L. *n* = 3.

We determined the cytotoxicity of stigmasterol on BEAS-2B cells by MTT (Figure 2(A)). Cell viabilities in different concentrations (0-40 μg/mL) indicated groups indicated that stigmasterol was nontoxic to BEAS-2B cells. BEAS-2B cells secrete a large number of inflammatory factors (IL-4, IL-5 and IFN-γ) after IL-13 stimulation (Figure 2(B)). Stigmasterol effectively reduced levels of the above inflammation cytokines in a dose-dependent manner. High-dose stigmasterol has a more significant inhibitory effect on inflammatory factors. Further, we also analyzed the changes in oxidative stress after dosages of stigmasterol by ELISA (Figure 2(C)) and flow cytometry (Figure 2(C)). IL-13 induction also increases the content of MDA, and decreases levels of CAT and SOD, which is significantly different from the control group. However, the level of MDA was downregulated, levels of CAT and SOD were upregulated after stigmasterol. Consistent with ELISA result, ROS content detected by flow cytometry in IL-13 group is higher than the control group. With the increase of stigmasterol dose, the content of ROS also decreases, but it was still higher than that of the control group.

### NK1-R is a potential target involved in the anti-inflammatory and antioxidant stress effects of this drug

**Figure 3.**
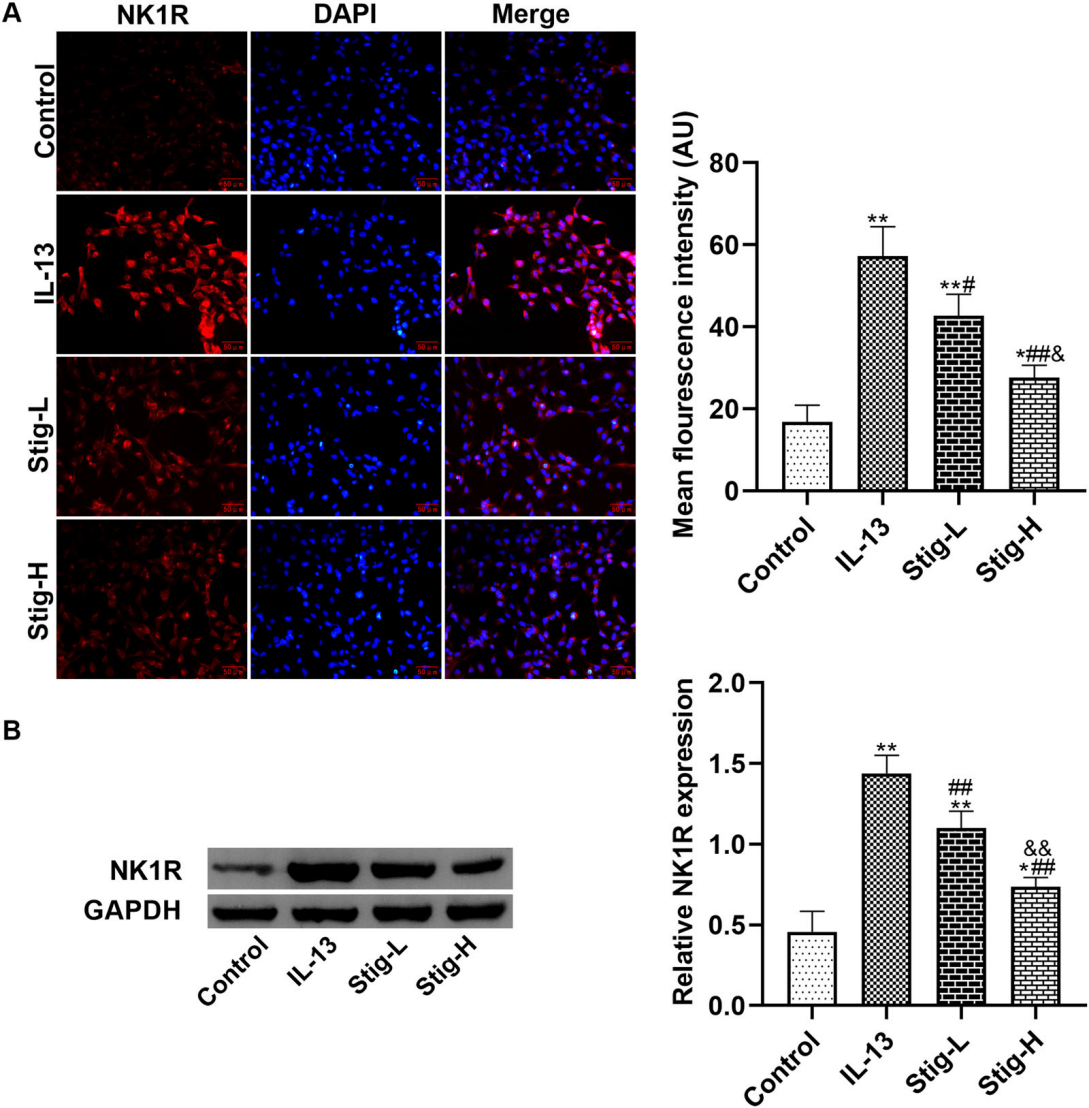
Stigmasterol downregulated the expression of NK1-R in IL-13-induced BEAS-2B cells. (A) We added two concentrations of stigmasterol into the medium, and observed the positive expression of NK1-R by immunofluorescence. Red: Cy3, blue: DAPI. Scale bar was 50 μm. Quantitative fluorescence intensity of NK1-R was analyzed by Image J 1.49p. (B) We also carried out western blot assay to quantify the expression of NK1-R. *p < 0.05, **p < 0.01, control; ^#^*p* < 0.05, ^##^*p* < 0.01, IL-13; ^&^*p* < 0.05, ^&&^ *p* < 0.01, Stig-L. *n* = 3.

Next, we further verified how stigmasterol regulates NK1-R expression by immunofluorescence staining (Figure 3(A)) and western blot (Figure 3(B)). First, we observed that NK1-R was expressed in the cytoplasm of BEAS-2B cells. After inflammatory stimulation, the expression of NK1-R in cells increased significantly with stronger fluorescence intensity. However, after two doses of stigmasterol, fluorescence intensity in Stig-L group and Stig-H group became weaker than that of IL-13 group, particularly in high doses of stigmasterol. Western blot showed similar results with immunofluorescence staining.

**Figure 4.**
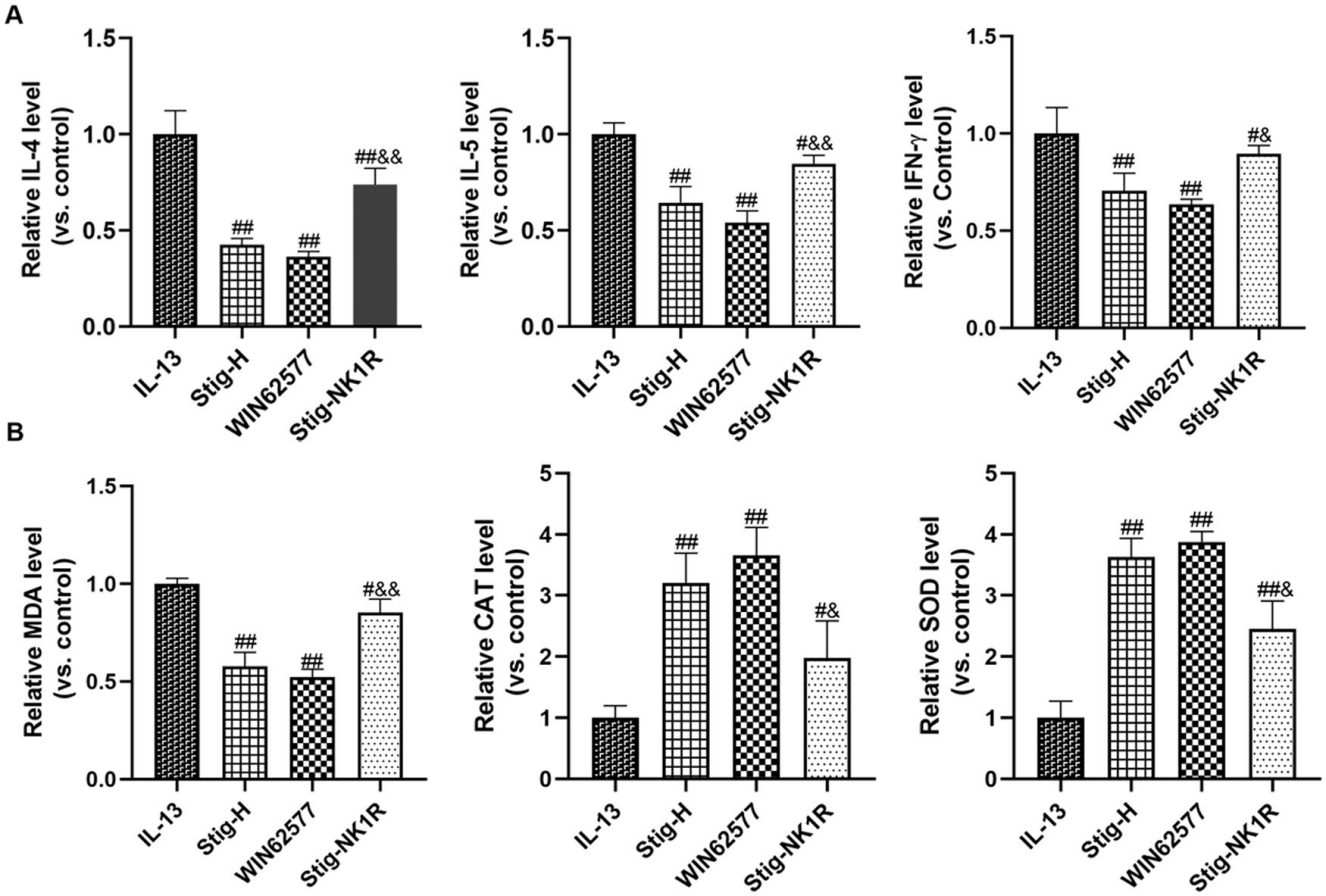
NK1R is a potential target of stigmasterol, which is involved in the anti-inflammatory (A) and antioxidant stress (B) effects of this drug. Inflammation cytokines (IL-4, IL-5 and IFN-γ) and oxidative stress markers (MDA, CAT and SOD) in cell supernatant were measured by ELISA methods. ^#^*p* < 0.05, ^##^*p* < 0.01, IL-13; ^&^*p* < 0.05, ^&&^*p* < 0.01, Stig-H. *n* = 3.

Further, we applied NK1R-specific antagonist WIN62577 and agonist recombinant human NK1R protein and assessed the levels of inflammation factors (Figure 4(A)) and oxidative stress indicators (Figure 4(B)) by ELISA. As seen from Figure 4(A), we observed a significant reduction of IL-4, IL-5 and IFN-γ after adding WNT62577 or stigmasterol. In order to further verify the downstream targeting effect of NK1-R, we added stigmasterol and NK-1R activator into a medium at the same time. Compared with Stig-H group, three cytokines significantly increased. For oxidative stress (Figure 4(B)), stigmasterol or WNT62577 could reduce the level of MDA, and increase the level of SOD and CAT. However, after combined supplementation of stigmasterol and recombinant NK1-R protein, these indicators also changed significantly compared with Stig-H group.

### Stigmasterol inhibited inflammation and oxidative stress in a mouse model of asthma by NK1-R

**Figure 5.**
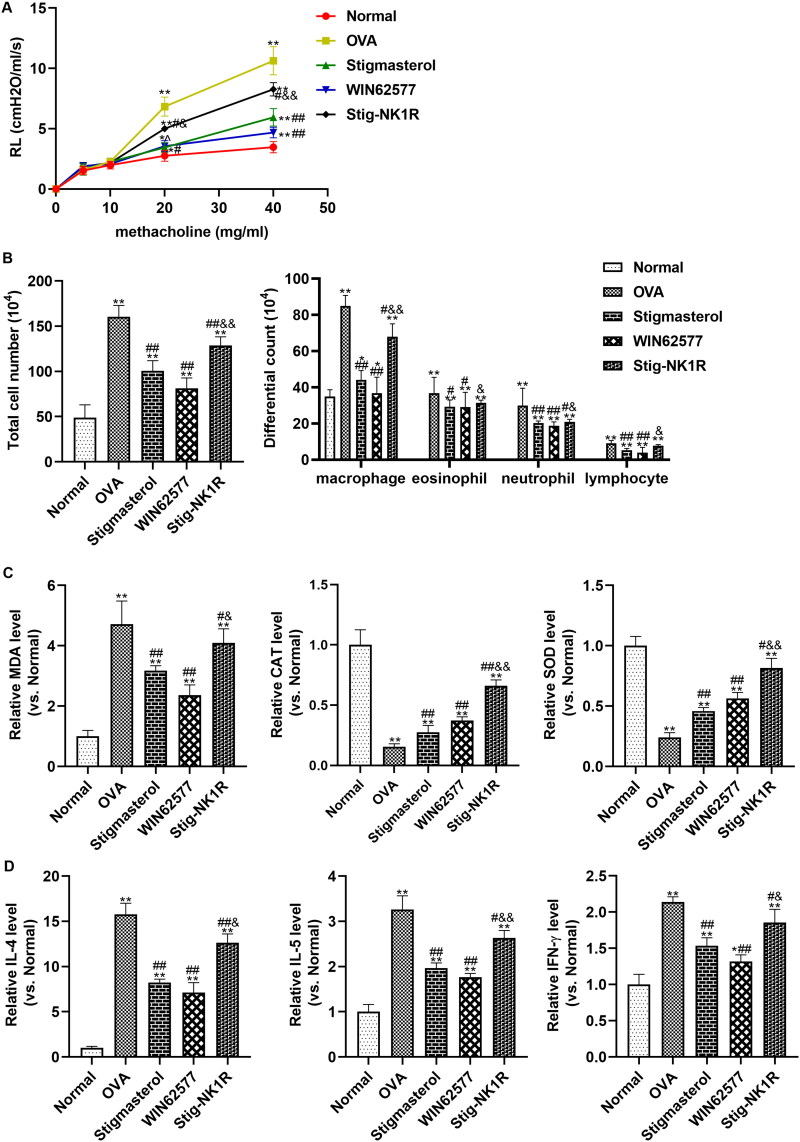
Stigmasterol inhibited the inflammation response and oxidative stress, and alleviated airway hyperreactivity by substance P receptor. (A) To evaluate airway hyperresponsiveness, invasive measures of dynamic lung resistance and compliance were measured by flexivent (SCIREQ, Emka Technologies) 2 days after the last drug injection. (B) Effect of stigmasterol on inflammatory cell count in BALF. Animals were sacrificed 24 h after the last administration, and BALF was collected for counts of eosinophil, lymphocyte, neutrophil, and macrophage. (C) Oxidative stress factors MDA, CAT and SOD were analyzed by an ELISA method. The experimental steps are strictly in accordance with the instructions of the kit. Experimental results normalized to normal group were showed in Y-axis of graphs. (D) Inflammatory cytokines IL-4, IL-5 and IFN-γ were analyzed by ELISA methods. Normalized results were also showed in graphs. *p < 0.05, **p < 0.01, normal; ^#^*p* < 0.05, ^##^*p* < 0.01, OVA; ^&^*p* < 0.05, ^&&^*p* < 0.01, Stigmasterol. *n* = 5.

To investigate the effect of stigmasterol on asthma *via* NK1-R, we constructed a mouse model of allergic asthma by OVA induction. For airway hyperreactivity, lung resistance was increased after OVA induction (Figure 5(A)). Intraperitoneal injection of stigmasterol or WNT62577 decreased lung resistance. But, lung resistance in Stig-NK1-R group showed an opposite trend after simultaneous administration of stigmasterol and recombinant NK1-R protein. In addition, after OVA induction, a significant increase in total cell number and immune cell infiltration (including macrophage, eosinophil, and neutrophil) were found in BALF fluid (Figure 5(B)). After treatment of stigmasterol or WNT62577, immune cells in BALF were significantly reduced. Meanwhile, oxidative stress parameters (Figure 5(C)) and inflammation factors (Figure 5(D)) in BALF fluid were also measured, to evaluate oxidative stress and inflammation factors in the context of asthma. Consistent with *in vitro* result, higher levels of MDA, IL-4, IL-5 and IFN-γ and lower levels of CAT and SOD were found in OVA group. The mentioned results indicated the important involvement of stronger oxidative stress and inflammation response in asthma. However, stigmasterol or antagonist WNT62577 contributed to the significant changes in above indicators (immune cell infiltration, oxidative stress markers, inflammation factors). Simultaneous administration of stigmasterol and WNT62577 partially offsets the inhibitory effect of stigmasterol on asthma-related indicators.

**Figure 6.**
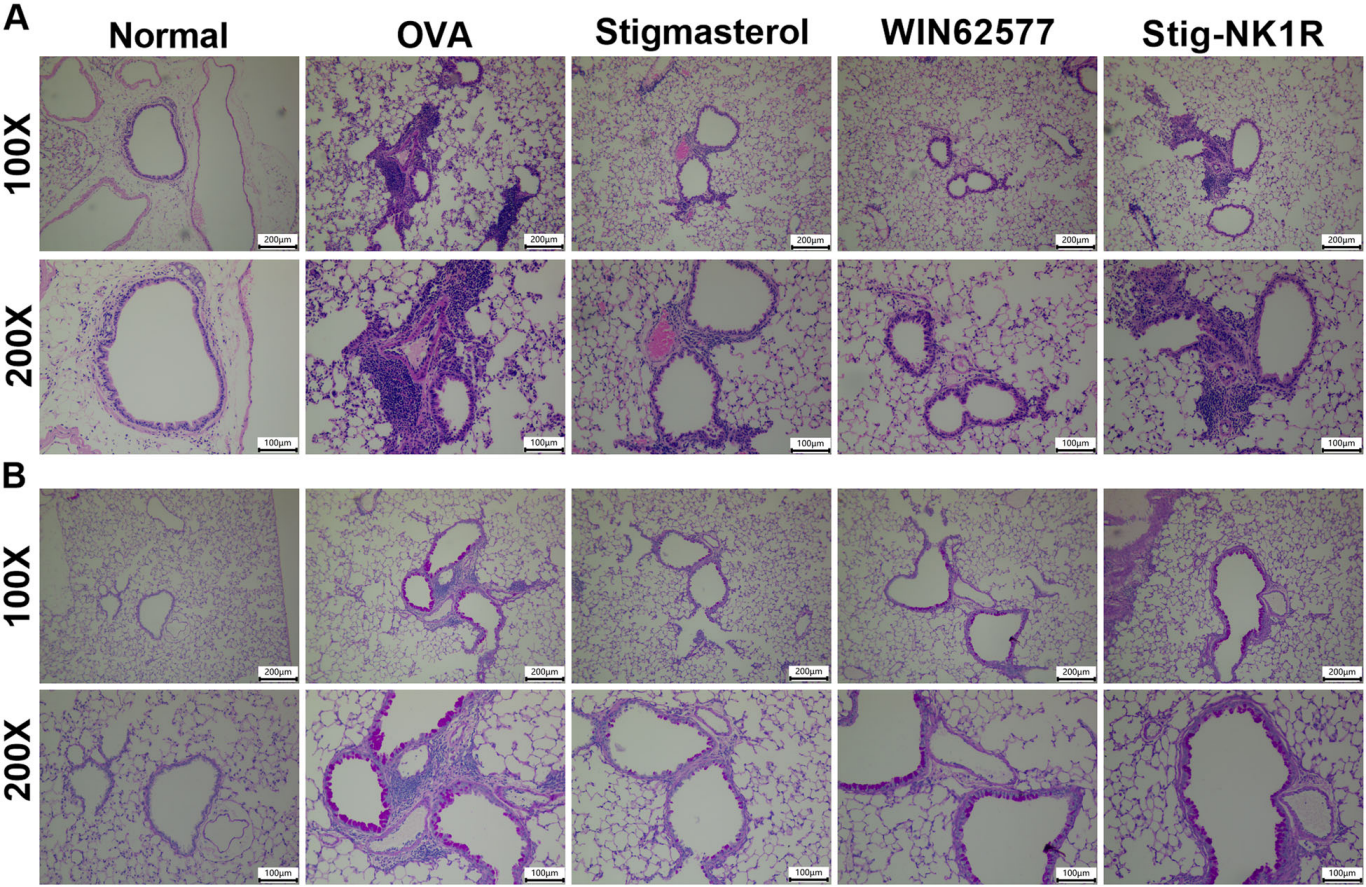
Stigmasterol improved inflammation infiltration and mucus hypersecretion in lung tissues by substance P receptor. Lung sections were stained with hematoxylin and eosin (H&E) staining (A) or periodic acid-Schiff reagent (PAS) staining (B). Micrographs depict representative tissues at 100× and 200× magnification.

Next, we performed HE (Figure 6(A)) and PAS (Figure 6(B)) staining to observe the inflammation infiltration and mucus secretion. Alveolar wall and bronchial of normal mice were clear without infiltration of inflammatory cells, and thin airway walls were visualized. After OVA challenge, we observed severe airway injury and a large amount of accumulation of inflammation cells around bronchioles. Mice treated with stigmasterol or WNT62577 displayed decreased peribronchial cellular infiltration of the lungs. However, increased immune cells in Stig-NK1R group were found. For mucus secretion, increased overproduction of mucus was found in asthma mice. Mice treated with stigmasterol or WNT62577 showed reduced mucus hypersecretion. However, compared with stigmasterol group, mucus secretion increased after recombinant NK1-R protein.

**Figure 7.**
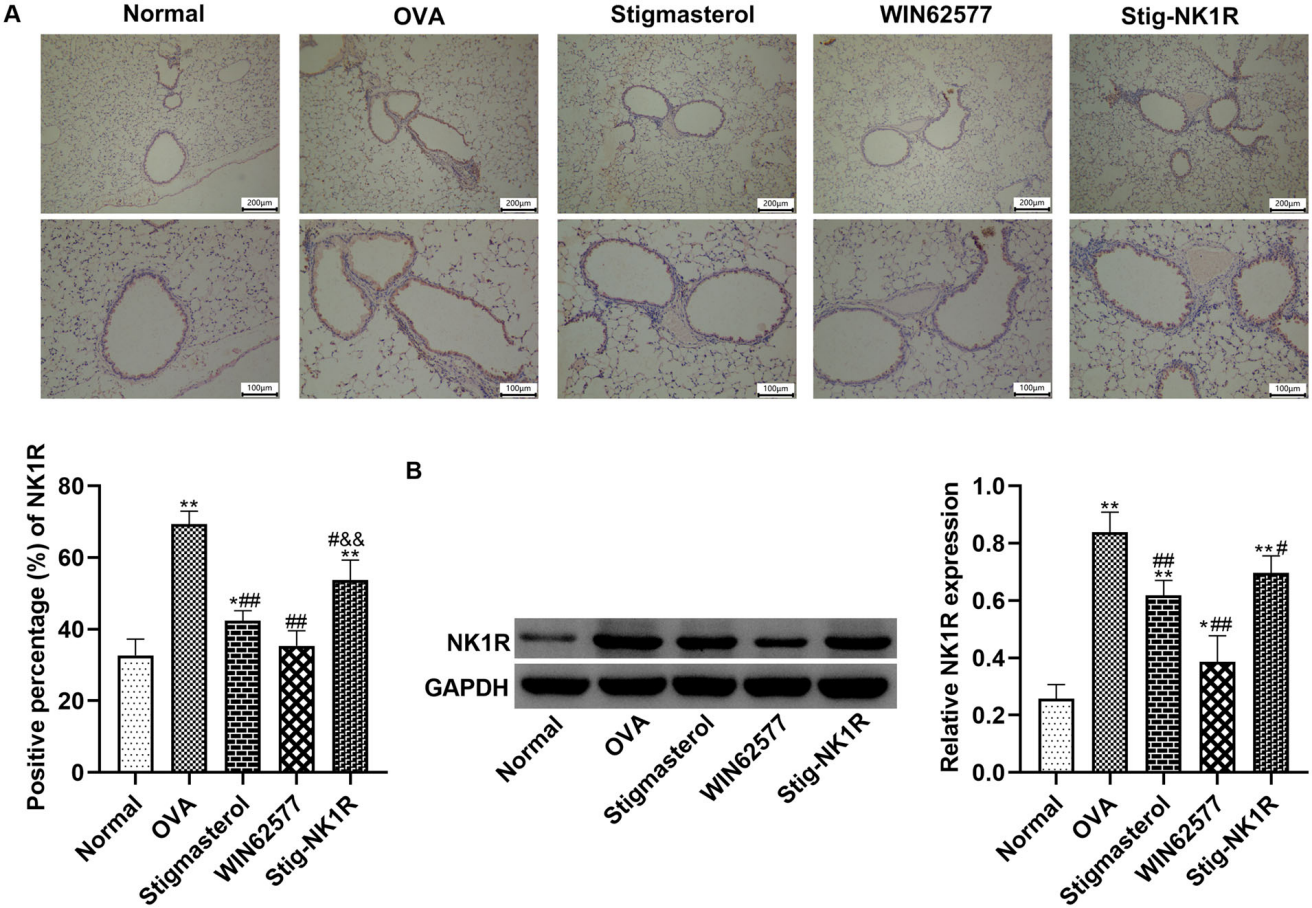
Stigmasterol decreased the expression of NK1-R in lung tissues of OVA rats by immunohistochemical staining (A) and western blot (B). Micrographs depict representative tissues at 100× and 200× magnification. These images were analyzed by Image J.49p. *p < 0.05, **p < 0.01, normal; ^#^*p* < 0.05, ^##^*p* < 0.01, OVA; ^&&^*p* < 0.01, Stigmasterol. *n* = 5.

Further, we analyzed NK1R expression in bronchioles in lung tissues by immunohistochemistry (Figure 7(A)) and western blot (Figure 7(B)). After OVA challenge, positive expression of NK1-R protein in lung tissues was higher than in other groups. Both stigmasterol and WNT62577 reduced the positive percentage of NK1R protein. But recombinant human NK1-R protein changes the expression of NK1-R, counteracting the downregulation of NK1-R by stigmasterol.

## Discussion

Substance P (SP) has been widely reported to take part in the pathophysiology of asthma (Chu et al. 2000; Hens et al. 2011; Farah and Salome 2012), which increases airway hyperreactivity and bronchoconstriction after allergen stimulation (Hens et al. 2011). Substance P is a neuropeptide composed of 11 amino acids, which is released from the nerve endings of many tissues, and its receptor neurokinin 1 (NK1-R) is highly expressed in the airways of the asthmatic body (Chu et al. 2000). SP binds to neurokinin 1 (NK1) receptor on the surface of effector cells. Substance P plays a crucial role in many inflammatory conditions, including asthma, arthritis and inflammatory bowel disease, etc. In the respiratory tract, NK1R can be found in the airway smooth muscle, submucosal gland and vascular endothelium, and some inflammatory cells also express NK1R (Mantyh 2002; Bright et al. 2018). NK has a strong effect on airway smooth muscle tension, bronchial circulation, airway secretion, and inflammatory and immune cells by activating neurokinin-1 (NK-1R) and neurokinin-2 receptor (NK-2R). Therefore, they are considered to play an important role in human respiratory diseases such as bronchial asthma and chronic obstructive diseases (Dinh et al. 2006). We used NK1R inhibitor WIN62577 to analyze the role of NK1R in asthma. The experimental results indicated that reduction of NK1R reduces inflammatory reaction and oxidative stress level in IL-13 induced BEAS-2B cells and asthma mice. SP has been demonstrated to affect the cellular redox states through activating neurokinin1receptor (NK1R) (Ebrahimi et al. 2022). Asthma inflammation may be manifested as an imbalance between oxidative stress and antioxidant defense. Oxidative stress contributed to airway inflammation response, leading to poor control of asthma and often severe acute attacks (Carpagnano et al. 2021). We also detected levels of oxidative stress markers (MDA, CAT and SOD), and frequent inflammation cytokines (IL-4, IL-5 and IFN-γ) in IL-13-induced BEAS-2B cells and BALF fluid of asthma mice. After OVA challenge, oxidative stress and inflammatory response became heavier, and increased NK1-R expression in bronchial species of lung tissue was found.

Stigmasterol is the main plant sterol in various herbs and has strong pharmacological activity, including anti-inflammation (Antwi et al. 2017; Feng et al. 2017; Morgan et al. 2021; Sampath, et al. 2021a; Sampath, et al. 2021b), anti-oxidative stress (Sun et al. 2019; Liang et al. 2020; Pratiwi et al. 2021), anti-allergic (Antwi et al. 2017), and so on. In the first part of the *in vitro* study, we demonstrated the protective effect of stigmasterol on IL-13-induced BEAS-2B cells and OVA-induced mice model of asthma with potent anti-inflammation and anti-oxidative stress effects. However, the relevant mechanism of stigmasterol in the treatment of asthma has not yet been established. At the initial design, we retrieved 34 asthma-related target molecules from TCMSP database. We analyzed the binding possibility between stigmasterol and 34 target molecules through molecular docking technology. Docking score in Table 1 shows that the binding force between stigmasterol and substance P receptor is the strongest. Therefore, we preliminarily judged that NK1-R was the potential target of stigmasterol. In the preliminary experiment, we have confirmed that two doses (10 and 20 μg/mL) are non-toxic to BEAS-2B cells. Through immunofluorescence and WB experiments, we concluded that stigmasterol downregulated NK1-R expression. So, can stigmasterol improve oxidative stress and inflammatory response in asthma by inhibiting NK1-R expression? This deserves our in-depth discussion. Therefore, NK1-R agonist (recombinant human NK1-R protein) and antagonist WNT62577 were applied. From experimental results, we found that stigmasterol inhibited oxidative stress and inflammation response in IL-13-induced BEAS-2B cells and asthma mice by NK1-R.

In brief, in this study, we draw several conclusions. 1) Stigmasterol displayed a protective role in asthma mice and *in vitro* BEAS-2B model, which is manifested with excellent effects of anti-inflammation and anti-oxidative stress. 2) NK1-R has been widely proven to be an important molecule in asthma. In this study, we confirmed that NK1-R is a potential target of stigmasterol, which is involved in the anti-inflammatory and antioxidant stress effects of this drug on asthma. In the future, we will continue to study the relevant mechanism of stigmasterol on asthma and further understand the therapeutic effect of stigmasterol on asthma. We will continue to focus on NK1-R as the molecular target of stigmasterol and carry out further in-depth research to explore downstream molecules and pathways.

## Data Availability

The datasets used and/or analyzed during the current study are available from the corresponding author on reasonable request.
